# Intermittent theta-burst stimulation with adjunctive D-cycloserine rapidly resolves suicidal ideation and decreases implicit association with death/suicide

**DOI:** 10.1017/S0033291724003313

**Published:** 2025-02-05

**Authors:** Myren N. Sohn, Jaeden Cole, Signe L. Bray, Alexander McGirr

**Affiliations:** 1 Department of Psychiatry, University of Calgary, AB, Canada; 2 Hotchkiss Brain Institute, University of Calgary, Calgary, AB, Canada; 3 Mathison Centre for Mental Health Research and Education, Calgary, AB, Canada; 4 Department of Radiology, University of Calgary, Calgary, AB, Canada

**Keywords:** D-cycloserine, death implicit association test, D-IAT, intermittent theta-burst stimulation, iTBS, major depressive disorder, MDD, NMDA receptor, repetitive transcranial magnetic stimulation, rTMS, suicidal ideation

## Abstract

**Background:**

Depressive disorders are the most common diagnosis among individuals who die by suicide, and intermittent theta-burst stimulation (iTBS) is a noninvasive treatment for those with difficult-to-treat depression who are at higher risk for suicide. Previous data suggests that pairing iTBS with D-cycloserine, a partial N-methyl-D-aspartate (NMDA) receptor agonist, improves antidepressant outcomes. However, its impact on suicide risk is not known.

**Methods:**

We examine suicidal ideation and implicit suicide risk after iTBS+D-cycloserine in two clinical trials (open-label trial [*n* = 12] and randomized placebo-controlled trial [RCT, *n* = 50]) involving adults with major depressive disorder and the acute effects of D-cycloserine on implicit suicide risk in a crossover trial (*n* = 18). Implicit suicide risk was assessed using the computerized death/suicide implicit association test (IAT), and depressive symptoms and suicidal ideation were assessed using the clinician-rated Montgomery–Asberg Depression Rating Scale (MADRS).

**Results:**

Open-label iTBS+D-cycloserine was associated with a rapid reduction in suicidal ideation, and iTBS+D-cycloserine was superior to iTBS+placebo in reducing suicidal ideation. Similarly, open-label iTBS+D-cycloserine was associated with decreased implicit suicide risk as measured by the death/suicide IAT, and iTBS+D-cycloserine was associated with greater decreases in death/suicide IAT scores compared to iTBS+placebo. A single acute dose of D-cycloserine in the absence of iTBS had no effect on implicit suicide risk.

**Conclusions:**

Adjunctive D-cycloserine with iTBS is a promising strategy to reduce suicidal ideation and implicit suicide risk in depression.

## Introduction

Suicide is a major public health problem and one of the leading causes of death worldwide (WHO, 2021). In the United States, the age-adjusted suicide rate is over 14 per 100,000 individuals, and suicide attempts outnumber deaths by suicide at a ratio of greater than 30:1 (Center for Disease Control and Prevention, 2023; National Center for Injury Prevention and Control, 2021). Prevention is critical to reducing deaths by suicide, and methods of assessing the risk of suicide have made great strides. The presence of psychopathology is one of the most robust risk factors, dwarfed only by a history of suicidal behavior (Nock et al., 2022; Nock, Hwang, et al., 2010). Accordingly, psychological autopsy studies consistently reveal the presence of a psychiatric condition in the period surrounding death by suicide in 90% of cases (Arsenault-Lapierre et al., 2004), with depressive disorders predominating in ~50% of cases (Arsenault-Lapierre et al., 2004; Bachmann, 2018; Holma et al., 2014). Although the majority of individuals with depression do not engage in suicidal behavior, some possess a vulnerability to suicidal behavior whether heritable, biological, or psychological and this vulnerability interacts with life adversity and mental illness in a diathesis-stress fashion to result in suicidal behavior (Mann & Arango, 1992; McGirr et al., 2008; Turecki & Brent, 2016). Yet, in the absence of means to specifically target this vulnerability, an individual’s risk for suicide remains largely yoked to their mental status.

Transcranial magnetic stimulation (TMS) is a noninvasive modality for targeting cortical circuits, and when synaptic plasticity-inducing patterns of stimulation are delivered TMS can be used to modify neural function (Lefaucheur et al., 2020). Major depressive disorder (MDD) is the noninvasive neurostimulation treatment modality with the largest evidence base, and an important treatment option for individuals who have not benefited from medications or psychotherapy, and therefore are at higher risk for suicide (Bergfeld et al., 2018; Lefaucheur et al., 2020; Nelsen & Dunner, 1995). As applied to suicidal ideation and suicide risk, there have been promising developments and successful applications of TMS to individuals with acute suicidal ideation (Cui et al., 2022; Hines et al., 2022; Li et al., 2024; Weissman et al., 2018).

The presumed therapeutic mechanism of action of repetitive TMS and newer protocols such as intermittent theta-burst stimulation (iTBS) involves inducing N-methyl-D-aspartate receptor-dependent synaptic plasticity in targeted circuits (Sohn et al., 2024). These activity-dependent changes are thought to resemble long-term potentiation (LTP) (Ziemann, 2004). However, it is well established that individuals with depression and those who die by suicide have molecular changes that constrain synaptic plasticity (Dwivedi, 2009, 2018; Dwivedi et al., 2003; Lutz et al., 2017; Pandey et al., 2008; Pandya et al., 2014; Turecki & Brent, 2016; Weder et al., 2014) and thus, may also constrain the therapeutic effects of iTBS. As such, pairing iTBS with a small molecule that helps facilitate iTBS-induced synaptic plasticity may improve its therapeutic efficacy. Indeed, we have recently shown that D-cycloserine, a partial NMDA receptor agonist, can enhance TMS-induced synaptic plasticity and metaplasticity in the healthy brain (Wrightson et al., 2023), rescue plasticity deficits in individuals with MDD (Cole et al., 2021), and lead to greater persistence of iTBS-associated plasticity (Cole et al., 2021). Accordingly, the combination of iTBS+D-cycloserine significantly improves antidepressant treatment outcomes as compared to iTBS+Placebo (Cole et al., 2022).

The effects of augmenting iTBS with D-cycloserine on suicidal ideation and suicide risk, however, are not known. Here, we examine clinician-rated suicidal ideation, as well as performance on the death/suicide implicit association test (death/suicide IAT), a test developed to index implicit bias for suicide (Nock, Park, et al., 2010) for which performance is associated with future suicide attempts (Barnes et al., 2017; Nock, Park, et al., 2010; Sohn et al., 2021; Tello et al., 2020). We do so by examining these secondary outcomes in two clinical trials pairing D-cycloserine with iTBS for the treatment of an acute major depressive episode. A third trial, a randomized placebo-controlled crossover study in a convenience sample of healthy individuals, tested the acute effects of D-cycloserine on implicit suicide risk as assessed using the death/suicide IAT.

## Methods

Between November 2019 and June 2023, three clinical trials were completed at the University of Calgary (NCT05081986, NCT03937596, and NCT05731323). All three studies were approved by the University of Calgary Ethics Board and Health Canada, and participants provided written informed consent. These studies followed the CONSORT guidelines. Primary outcomes for two of the trials have been previously published (Cole et al., 2022; Wrightson et al., 2023).

### Participants

Recruitment occurred through advertisements and referrals. General eligibility criteria for all three trials included the following: males and females aged 18–65, medically stable, and free of TMS safety concerns, including a history of epilepsy, stroke, intracranial mass-occupying lesions, Huntington’s disease, or traumatic brain injury with a loss of consciousness exceeding 5 min. Participants who were female at birth could not be pregnant or lactating and committed to contraception or abstinence during the trial.

Two of the trials (*n* = 12 open-label NCT05731323 and *n* = 50 randomized placebo-controlled NCT03937596) involved individuals with an acute major depressive episode. For these individuals, the inclusion criteria were depressive symptoms of at least moderate severity (as defined by ≥18 on the Hamilton Depression Inventory (HAMD-17)) and having failed between 1 and 3 adequate trials of antidepressant medication. These participants had normal blood work in the past month, passed the TMS adult safety screening (TASS) questionnaire, and had not previously failed a course of TMS. Participants for these outpatient trials were not actively suicidal as defined by a score ≥ 4 on the suicide item of the Montgomery–Asberg Depression Rating Scale (MADRS).

A convenience sample of *n* = 20 healthy, nondepressed, and nonsuicidal individuals was recruited as part of a randomized placebo-controlled crossover study where participants received a single dose of D-cycloserine in the absence of iTBS to the dorsolateral prefrontal cortex (NCT05081986).

### Adjunctive D-cycloserine

D-cycloserine (Seromycin) was purchased in 250 mg capsules (Parsolex GMP Center, West Lafayette, IN). The open-label study was designed to test weight-based dosing (25 mg D-cycloserine per 17.5 kg body weight), whereas the randomized placebo-controlled trial (RCT) and healthy nondepressed sample utilized 100 mg capsules. Placebo capsules contained 100 mg or 25 mg per 17.5 kg body weight of microcrystalline cellulose and could not be differentiated from D-cycloserine capsules.

Participants were given their study medication at enrollment and instructed to ingest the medication 60–120 min prior to each treatment. In the open-label study, medication was paired with stimulation for 4 weeks. For the placebo-controlled RCT, study medication was paired with iTBS for the first 2 weeks of the 4-week trial.

### Intermittent Theta-burst stimulation

iTBS was administered using the MagPRO X100 system with the Cool-B70 coil (MagVenture, Denmark) in combination with Visor 2 neuronavigational software. The left DLPFC stimulation site was determined using the Beam F3 method (Beam et al., 2009). The iTBS protocol consisted of 20 trains of 10 50 Hz triplets, delivered at 5 Hz, with 8 s intertrain interval, for a total of 600 pulses at 80% of the participant’s resting motor threshold (RMT).

The RMT was determined by placing electrodes on the first dorsal interosseous muscle (FDI). The RMT was defined as the lowest stimulator intensity to give 5/10 motor-evoked potentials with >50 uV amplitude.

iTBS treatments took place Monday to Friday, for 4 weeks in both the open-label trial and the randomized placebo-controlled trial.

### Clinical assessments

All assessments were conducted by a blinded study psychiatrist. Suicide histories were characterized in a clinical interview using the Columbia Suicide Severity Rating Scale [CSSRS; (Posner et al., 2011)]. Depressive symptoms were assessed using the clinician-rated MADRS (Montgomery & Asberg, 1979). Item 10 of the MADRS was used to measure clinician-rated suicidal ideation in the past week. This item ranges from 0- ‘enjoys life or takes it as it comes’ to 6- ‘explicit plans for suicide when there is an opportunity/active preparation for suicide’.

### Death/suicide implicit association task

Participants completed the death/suicide IAT, a computerized task that was developed to leverage the differential strength of association of life/death with the self as a measure of implicit suicide risk (Nock, Park, et al., 2010). At the top of the screen are ‘targets’ (Death vs Life) alongside the self as an attribute (Me vs Not Me) to which participants must assign a stimulus word in each trial. Beginning with training for target category and attribute sorting, the task is then composed of four blocks for a total of 120 trials. The test generates a summary score, the *D*-score, that consists of the reaction time difference between ‘Me-Death’ and ‘Not Me-Life’ pairings relative to ‘Me-Life’ and ‘Not Me-Death’ pairings. Negative *D*-scores represent a stronger association between ‘Me-Life’ and ‘Not Me-Death’. Depressed participants completed the death/suicide IAT at baseline and after 2 weeks (RCT) or 4 weeks (open-label) of daily iTBS treatments, and healthy participants completed the task 2 h after ingesting either a single dose of placebo or D-cycloserine and then again 1 week later in a crossover design.

### Statistical analyses

All statistical analyses were performed using IBM SPSS Statistics 29. Demographics and baseline clinical characteristics were analyzed using independent samples *t*-tests for continuous and Chi-Squared tests for categorical variables. Histograms and the Shapiro–Wilks test were used to assess normality. If the normality assumption was violated, a Mann–Whitney test was performed. Alpha was set at 0.05.

Reporting of results follows the same order for both clinician-rated suicidal ideation and death/suicide IAT: (1) open-label iTBS+D-cycloserine in participants with MDD, (2) randomized placebo-controlled iTBS+dycloserine in participants with MDD, and (3) single-dose placebo-controlled crossover study in the absence of iTBS in healthy participants.

#### Clinician-rated suicidal ideation

For open-label iTBS+D-cycloserine data, a Friedman’s test was conducted to assess change in clinician-rated suicidal ideation from baseline to posttreatment (MADRS item 10). Post hoc Wilcoxon signed rank tests were conducted to assess differences in suicidal ideation compared to baseline at each time point.

In the placebo-controlled RCT, intention-to-treat analyses (ITT) with the last observation carried forward were conducted to determine the interaction between the treatment group and change in clinician-rated suicidal ideation after 2 and 4 weeks of treatment. Specifically, separate ordinal logistic regressions were conducted to assess the effect of the group on item 10 of the MADRS. Analyses were conducted with and without a percent change in nonsuicide depressive symptoms as a covariate (first nine items of the MADRS).

Suicidal ideation was not present in healthy controls participating in the single-dose placebo-controlled crossover study.

#### Death/suicide IAT

Open-label iTBS+D-cycloserine data were analyzed using a repeated-measures within-subjects (TIME: Pre, Post) analysis of variance (ANOVA) to assess change in death/suicide IAT *D*-scores from baseline to posttreatment. A separate repeated-measures within-subjects analysis of covariance (ANCOVA) was then conducted controlling for the percent change in nonsuicide depressive symptoms.

Placebo-controlled iTBS+D-cycloserine RCT data was analyzed using ITT analyses with the last observation carried forward and analyses were repeated using generalized linear mixed effects models (GLMEM with identity link function) to better account for missing data. Analyses included a between-subjects (TREATMENT: D-Cycloserine, Placebo) repeated-measures (TIME: Pre, Post) ANOVA to compare changes in death/suicide IAT *D*-scores in placebo and D-cycloserine groups. Analyses were repeated controlling for percent change from baseline to posttreatment in clinician-rated nonsuicide depressive symptoms.

For the single-dose placebo-controlled crossover study, a paired samples *t*-test was conducted to compare death/suicide IAT performance with and without D-cycloserine. To assess potential order effects, we used an independent samples *t*-test comparing death/suicide IAT performance in individuals who received D-cycloserine or placebo during the first study session.

## Results

Participant characteristics and baseline clinical characteristics for participants in the three studies are presented as separate panels in Table 1. The samples were comparable with respect to clinical and demographic features, and 27.4% of those with MDD had previously attempted suicide (*n* = 4/12 open-label participants and *n* = 13/50 RCT participants). Primary clinical outcomes of the RCT have been reported elsewhere (Cole et al., 2022). In the open-label study, all participants completed 4 weeks of open-label treatment. In the RCT, three participants withdrew prior to repeating the death/suicide IAT. In the single-dose crossover trial, two data points were lost due to computer malfunction, and complete death/suicide IAT data were available for *n* = 18 participants.

**Table 1.** Participant characteristics

Characteristic	Trial			
Population	Major depressive disorder			Healthy
Design	Open-Label	RCT		Crossover
	*n* = 12	Placebo (*n* = 25)	DCS (*n =* 25)	*n* = 18
Age, mean (SD), y	37.42 (15.93)	40.88 (13.36)	40.64 (13.64)	34.0 (9.89)
Sex, % female	66.7%	60.0%	64.0%	33.3%
Ethnicity, % European origins	91.7%	80.0%	68.0%	77.8%
Handedness, % right	66.7%	100.0%	80.0%	88.9%
Education, mean (SD), y	14.08 (4.96)	16.44 (2.10)	16.44 (4.16)	-
Number of depressive episodes, mean (SD)	7.33 (5.42)	8.65 (7.61)	7.74 (11.76)	-
MADRS, mean (SD)	31 (5.27)	30.32 (4.24)	30.44 (4.35)	-
MADRS item 10, mean (SD)	1.67 (1.07) Range: 0–3	1.72 (1.14) Range: 0–3	1.92 (0.76) Range: 0–3	-
History of suicide attempt	33.33%	20.0%	32.0%	-
Mean number of previous SAs, range	0.83 Range: 0–5	0.45 Range: 0–6	0.68 Range: 0–5	-
Baseline Death/suicide IAT scores, mean (SD)	−0.30 (0.30)	−0.45 (0.38)	−0.45 (0.42)	Placebo: −0.61 (0.40) D-Cycloserine: −0.61 (0.37)

DCS, D-cycloserine; RCT, randomized controlled trial; MADRS, Montgomery-Asberg Depression Rating Scale; Death/suicide IAT, death/suicide implicit association test; SD, standard deviation.

At baseline, 14.5% of participants with MDD (*n* = 2/12 open label and *n* = 7/50 RCT) scored ≥0 on the death/suicide IAT, representing stronger associations of the self with death as compared to life (*n* = 3/18 healthy controls; Table 1). In both the open-label and RCT samples, previous suicide attempters had higher scores on the death/suicide IAT suggesting a stronger association of the self with death; however, this was not statistically significant (open-label: *M* = −0.08, SD = 0.23 versus *M* = −0.41, SD = 0.28, *t*(10) = −2.06, *p* = 0.07; RCT *M* = −0.31, SD = 0.34 vs *M* = −0.50, SD = 0.40, *t* (48) = −1.55, *p* = 0.13). The magnitude of this difference is consistent with a small effect size as reported in a recent meta-analysis of the death/suicide IAT and previous suicidal behavior (Sohn et al., 2021).

### Clinician-rated suicidal ideation

#### Open-label iTBS + D-cycloserine trial

**Figure 1.**
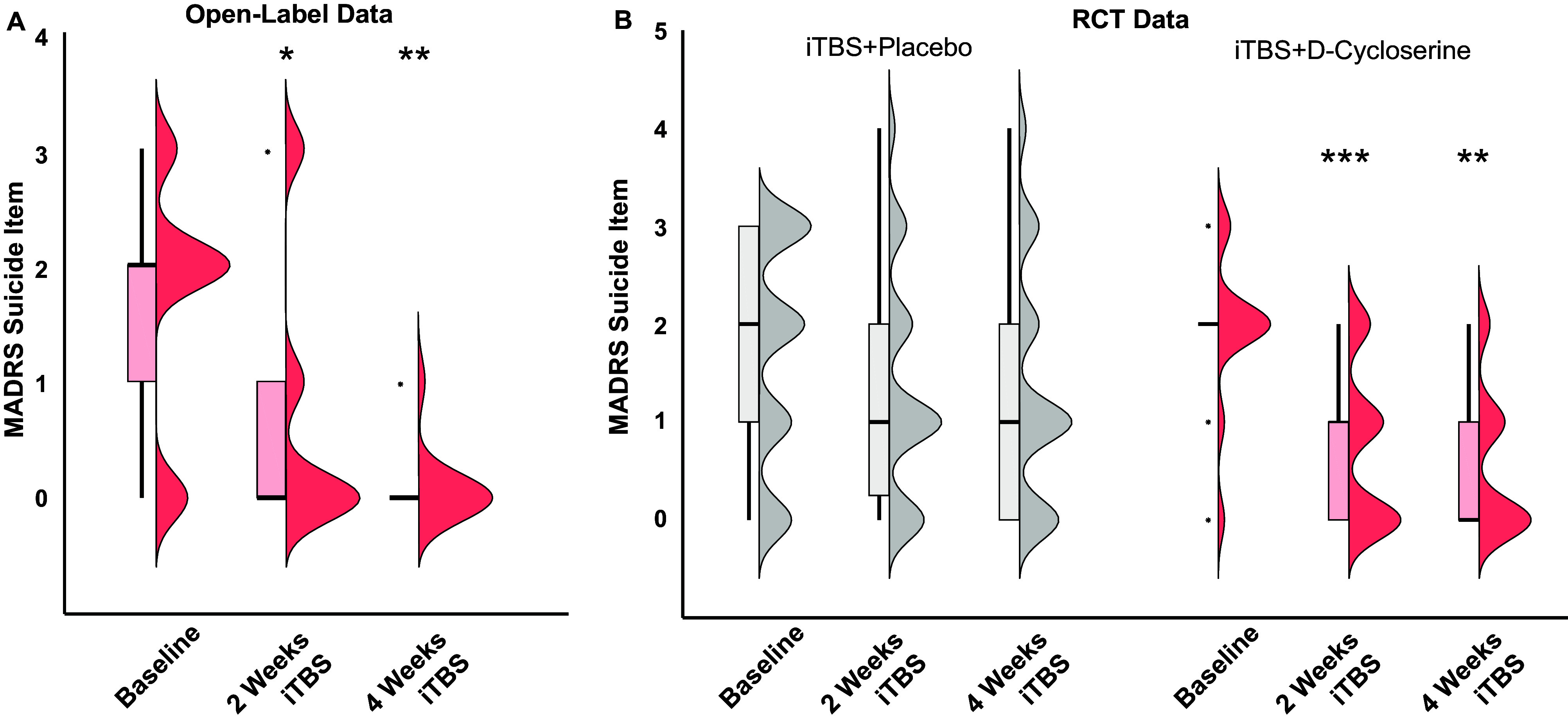
Clinician-rated suicidal ideation. Half-violin plots illustrating the distribution and density of the Montgomery-Asberg Depression Rating Scale suicidal ideation item over time in (a) a 4-week open-label trial of iTBS+D-cycloserine and (b) a 4-week RCT of iTBS+Placebo versus iTBS+D-cycloserine. *p < 0.05; **p < 0.01; ***p < 0.001.

Open-label adjunctive D-cycloserine with iTBS was associated with a decrease in the clinician-rated MADRS suicidal ideation item (Friedman Test (χ^2^(2,12) = 9.24, *p* = 0.01; Figure 1a). Post-hoc Wilcoxon signed rank tests revealed a statistically significant reduction in suicidal ideation from baseline to treatment week 2 (*Z* = −2.07, *p* = 0.039) and treatment week 4 (*Z* = −2.75, *p* = 0.006).

#### Randomized placebo-controlled iTBS + D-cycloserine trial

In the RCT, compared to the iTBS+Placebo condition, participants receiving adjunctive iTBS+D-cycloserine had a larger reduction in suicidal ideation after both 2 weeks (Nagelkerke pseudo *R*^2^ = 0.157, Estimate = −1.51, SE = 0.56, Wald = 7.20, *p* = 0.007) and 4 weeks (Nagekerke pseudo *R*^2^ = 0.126, Estimate = −1.33, SE = 0.55, Wald = 5.99, *p* = 0.01; Figure 1b). This effect remained statistically significant when controlling for percent change in nonsuicide MADRS depressive symptoms after 2 weeks of treatment (Nagelkerke pseudo *R*^2^ = 0.249, Estimate = −1.30, SE = .57, Wald = 5.19, *p* = 0.023) but not after 4 weeks of treatment (Nagelkerke pseudo *R^2^* = 0.226, Estimate = −0.798, SE = .58, Wald = 1.87, *p* = 0.172) suggesting that improvements in suicidal ideation occurred prior to improvements in other depressive symptoms.

#### Single-dose randomized placebo-controlled crossover trial without iTBS

None of the healthy participants had suicidal ideation and therefore we could not test the effects of a single dose of D-cycloserine relative to a placebo on suicidal ideation.

### Death/suicide IAT

#### Open-label iTBS + D-cycloserine trial

**Figure 2.**
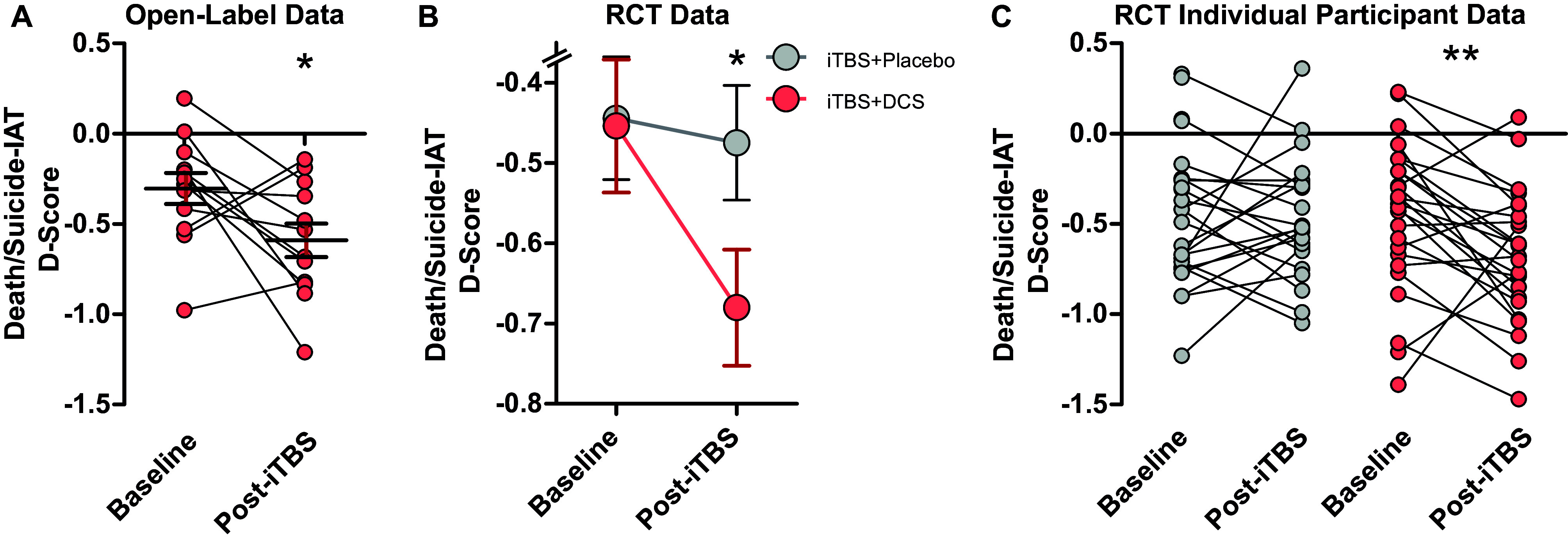
The death/suicide implicit association test *D*-score. (a) Open-label group and individual participant data are presented at baseline and after 4 weeks of iTBS+D-cycloserine. Randomized placebo-controlled trial group change in *D*-Scores are presented in (b) and individual participant data are presented in (c). *p < 0.05; **p < 0.01; ***p < 0.001.

Open-label adjunctive D-cycloserine with iTBS resulted in a statistically significant reduction in death/suicide IAT D-scores (*F*(1,11) = 4.85, *p* = 0.05, partial eta^2^ = 0.31) from baseline (*M* = −0.30, SD = 0.30) to treatment end (*M* = −0.59, SD = 0.32; Figure 2a). The two participants who scored >0 on the death/suicide IAT at baseline scored below zero at the end of treatment (Mean difference = −0.68, SD = 0.31).

#### Randomized placebo-controlled iTBS + D-cycloserine trial

In the placebo-controlled RCT, there was no significant difference at baseline in death/suicide IAT performance between iTBS+Placebo and iTBS+D-cycloserine treatment groups (*t* (48) = 0.08, *p* = 0.93; Table 1). We observed a statistically significant TREATMENT × TIME interaction (*F* (1,48) = 5.60, *p* = 0.02, partial eta^2^ = 0.11; Figure 2b) in which participants in the iTBS+D-cycloserine group had significantly larger decreases in death/suicide IAT *D*-scores than participants in the iTBS+Placebo condition, reflecting a strengthening of association of the self with life. This interaction remained statistically significant when controlling for percent change in nonsuicide depressive symptoms (*F*(1,47) = 4.08, *p* = 0.049, partial eta^2^ = 0.08; MADRS items 1–9). Individual participant data (Figure 2c) illustrates this decrease in death/suicide IAT scores after treatment in the iTBS+D-Cycloserine group. To better account for missing data, this analysis was repeated using a GLMEM, which revealed a trend toward a significant TREATMENT × TIME interaction (*F*(2, 93) = 2.97, *p* = 0.056).

We also examined the change in death/suicide IAT performance for the *n* = 7 individuals who scored ≥0 at baseline. Five of these participants completed the death/suicide IAT at baseline and posttreatment. For all five participants, death/suicide IAT performance improved from baseline to posttreatment (iTBS+D-Cycloserine: *n* = 3, Mean difference = −0.41, SD = 0.19; iTBS+Placebo: *n* = 2, Mean difference = −0.34, SD = 0.04; Figure 2c).

#### Single-dose randomized placebo-controlled crossover trial without iTBS

To determine whether D-cycloserine or placebo in the absence of iTBS impacts performance on the death/suicide IAT, we administered the death/suicide IAT to a convenience sample of *n* = 18 healthy individuals taking part in a single-dose crossover trial. This revealed test–retest validity (*r* = 0.58), no order effect (*t*(36) = −0.46, *p* = 0.65), and no effect of *D*-cycloserine on death/suicide IAT *D*-scores compared to placebo (*t*(17) = −0.004, *p* = .997).

## Discussion

Our open-label and randomized placebo-controlled data suggest that adjunctive D-cycloserine with iTBS leads to a rapid reduction in clinician-rated suicidal ideation and strengthens implicit associations with life on the death/suicide IAT. These findings are important because although decreasing suicidal ideation targets proximal risk, implicit suicide risk as measured by the death/suicide IAT is associated with an increased risk of suicide attempt within 6 months (Sohn et al., 2021). As such, iTBS+D-cycloserine may modify an underlying vulnerability for future suicidal behavior in addition to resolving current risk. Moreover, a strength of the study is that depressed participants without suicidal ideation were retained in analyses, allowing us to test and rule out the possibility of emergent suicidal ideation during treatment.

The therapeutic effects of iTBS are thought to be mediated by NMDA-dependent synaptic plasticity (Hallett, 2007), thus deficits in plasticity in MDD may limit its physiological effect (Cole et al., 2021; Kuhn et al., 2016; Player et al., 2013; Vignaud et al., 2019). Based on physiological data in the motor cortex showing that iTBS+D-cycloserine normalizes and stabilizes synaptic plasticity in MDD (Cole et al., 2021), we hypothesized that this combination would result in greater antidepressant efficacy, including suicidal ideation. Nevertheless, the rapid reduction in suicidal ideation we observed over and above reductions in the depressive syndrome was unexpected, with the caveat that the severity of suicidal ideation in participants was low. Given the clinical importance of suicide and the scalability within existing TMS infrastructure, adjunctive D-cycloserine with iTBS is highly deserving of additional research. If the antisuicidal effects of iTBS+D-cycloserine are confirmed, this would be clinically significant.

The ability of iTBS+D-cycloserine to change not only suicidal ideation but also implicit associations contrasts with other rapid-acting antisuicidal interventions. For example, subanesthetic ketamine rapidly reduces suicidal ideation for up to 72 h (Witt et al., 2020; Xu et al., 2016), an effect that may be maintained using high doses of D-cycloserine (Chen et al., 2019). However, this intervention results in no change to performance on the death/suicide IAT (Lamontagne et al., 2024; Price et al., 2009; Price et al., 2014). Indeed, the death/suicide IAT is a relatively stable metric that measures an element of suicide risk not captured in typical suicide assessments (Brent et al., 2021; Glenn et al., 2019; Nock, Park, et al., 2010) and may serve as a means of identifying individuals for suicide prevention measures (Sohn et al., 2021). However, it remains to be determined whether a change in performance on the death/suicide IAT is associated with a change in future suicidal behavior.

Evidence suggests that circuit-level remodeling may underlie the therapeutic effects of TMS treatments (Fox et al., 2012, 2013; Siddiqi et al., 2020, 2021, 2022) and this has relevance to suicidal ideation and the death/suicide IAT. Specifically, the anticorrelation between the left DLPFC target site and the subgenual anterior cingulate cortex (ACC) is an important predictor of therapeutic efficacy of TMS for depression (Fox et al., 2012), and neuroimaging studies in individuals with suicidal thoughts and behaviors have implicated the ACC in suicidal ideation (Schmaal et al., 2020). Similarly, both the DLPFC and the ACC have been implicated in implicit associations with life (Ballard et al., 2019). We speculate that iTBS+D-cycloserine-driven changes in DLPFC-ACC circuitry could provide a basis for decreased suicidal ideation and strengthened implicit associations with life.

### Limitations

All three trials are limited by their sample sizes and were conducted at a single center. Second, these outpatient trials did not include individuals at acute risk for suicide, and therefore, these findings must be confirmed in individuals with active suicidal ideation. Third, although death/suicide IAT performance is associated with future suicidal behavior, it has not yet been demonstrated that an intervention changing death/suicide IAT performance has a subsequent impact on the trajectory of suicidal behavior. Lastly, we utilized a single IAT, and therefore, it is unclear whether changes to the death/suicide IAT are specific to the strengthening of an association of the self with life, and it is possible that iTBS+D-cycloserine produces changes to other implicit associations relevant to depression and suicide (Price et al., 2022).

## Conclusions

iTBS+D-cycloserine reduces suicidal ideation and decreases implicit suicide risk as measured by the death/suicide IAT. These promising antisuicidal findings require independent replication and extension to acutely suicidal individuals.
